# A career reflection: lifetime achievements in avian neuroanatomy and physiology

**DOI:** 10.3389/fphys.2026.1779850

**Published:** 2026-03-13

**Authors:** Wayne J. Kuenzel

**Affiliations:** Department of Poultry Science, University of Arkansas, Fayetteville, AR, United States

**Keywords:** academic support, chickens, food intake, funding, reproduction, sabbatical leaves, stress

## Background and past education

My parents valued education and made sure all three of their children attended college. After graduation from Bucknell University with a B.S. degree in Biological Sciences, I received an offer to attend graduate school and received an M.S. degree from Bucknell University and a PhD from the University of Georgia (UGA). My mentor was Dr. Carl W. Helms who obtained his PhD from Harvard University. His major advisor was Professor Ernst Mayr, regarded as one of the greatest evolutionary biologist of the 20th century. Dr. Helms studied the complete annual cycles of migratory birds that wintered in different areas of the south and central U.S. and migrated to Canada to breed. I focused on investigating the annual cycle of the White-throated Sparrow, *Zonotrichia albicolis* and utilized a surgical procedure that removed targeted neurons from the brain of living birds. I addressed the hypothalamus and found that surgically removing a small structure at the base of the hypothalamus, the ventromedial hypothalamic nucleus, resulted in disruption of a bird’s annual cycle. Specifically, the procedure caused obesity that mimicked the remarkable weight gain migratory birds display every spring to store up fat reserves prior to their rigorous flight to their breeding grounds in Canada. The same surgical procedure also significantly increased water consumption. Unexpectedly, the operation inhibited gonadal development and molting of feathers and completely terminated a specific behavior during the nighttime called *Zugunruh*e. The German term applies to migratory birds, when maintained in cages designed to record motor activity patterns, particularly hopping from one perch to another throughout a typical 24-h day. Nocturnal migratory birds will display robust motor activity at night, during the spring and fall when they would initiate their flight to their breeding grounds in spring and their return flight to their wintering grounds in the southern U.S. The increased nocturnal activity is regarded as equivalent to a bird’s desire to migrate. It was the first time in a migratory bird that removal of a specific hypothalamic structure resulted in obesity and the loss of specific behavioral processes characteristic of the annual cycle of migratory birds (Kuenzel and Helms, 1967; 1970; 1974; Kuenzel, 1974). Upon completing a PhD at UGA, my major professor encouraged me to pursue an academic career. I still remember his kind words.

An important obligation remained. I was in ROTC at Bucknell University. I therefore entered the U.S. Army for 2 years and was sent to the Medical Service Corps (MSC), Ft. Sam Houston, TX. I and others served as military instructors and taught human anatomy and physiology to soldiers. Soldiers remaining at Ft. Sam Houston would be trained as combat medics. During the second year at the MSC, at Ft. Sam, I was asked to serve as Chief, Basic Science Branch and was promoted from first lieutenant to captain. Thereafter, I obtained a National Institutes of Health (NIH) Postdoctoral Fellowship and spent 3 years at Cornell University experimenting with chickens under the direction of the neuroendocrine expert, Dr. Ari van Tienhoven. Additionally, the positive experience in the laboratory of Dr. Ari van Tienhoven, encouraged me to apply for a faculty position and was fortunate to spend half of my career (26 years) in the Poultry Science (POSC) Department (Dept.) at the University of Maryland, College Park, MD and am currently working in the POSC Dept. at the University of Arkansas, Fayetteville that began in 2000.

### Academic roles at the Univ. of Maryland (UMD) and Univ. of Arkansas, fayetteville (UAF)

At both the UMD and UAF, I was hired primarily to (a) produce original research and (b) teach courses (three courses at UMD, two courses at UAF). Additionally, a third role was to serve on committees, from international ones down to university committees. The order of importance for the 3 roles at both UMD and UAF was to build a reputation in a particular research area, followed by teaching and service on committees. In research, I have focused on the avian brain, specifically its anatomy and physiology. What was so valuable at both institutions was the opportunity to teach courses in the disciplines that were relevant to my research interests. The major academic discipline I wished to address was an overall knowledge of avian neuroendocrine physiology. The specific research area was to develop knowledge of neural structures involved with the anterior pituitary gland. This would include contributing new, anatomical and functional knowledge of the regulation of specific neuroendocrine processes. At both UMD and UAF, I have contributed new knowledge to three avian neuroendocrine areas: 1) the location of structures regulating the reproductive system, 2) stress pathways and 3) seasonal regulation of food intake.

### Value of (1) academic support at UMD/UAF, (2) two group meetings, (3) sabbatical years, (4) funding

There were four processes/events experienced that enabled me to contribute a few advancements associated with avian brain function: 1) Academic structure and support at UMD and UAF provided support for a technical assistant person, 2) two group meetings that focused on avian neuroanatomy, 3) sabbatical leaves were encouraged by UMD and UAF, 4) both universities have an Agricultural Experiment Station that has an annual, competitive grants program providing funding for research programs involving crops, and/or agricultural animals designed to improve food production or agribusiness. Success in this program has provided opportunities for faculty to be successful in national grants programs due to the successful data collection from a previous experiment station grant. Funding I received was also used to support presentations at scientific meetings and publications in journals. Additionally, one objective throughout my career was to train graduate students to become knowledgeable and comfortable in pursuing a specific research area and develop an ability to communicate their research results not only to specific scientific audiences, but also to their local public community when appropriate.

Note that the four processes/events emphasized previously were not sequential. In fact, all four were interactive throughout my career. In my chosen research field, avian neuroanatomy, I have always been intrigued by determining the specific location of structures within the brain, finding their appropriate name and abbreviation, their product and receptors, and critical tests to complete for demonstrating their specific function/s.

During my career, I was invited to join two groups of scientists. There was a significant overlap of specific individuals in different named groups. The first one was the Thinktank, organized by Dr. Anton (Tony) Reiner at the University of Tennessee Health Science Center, Memphis, TN. Years later, the Nomenclature Forum was initiated by Dr. Eric Jarvis, Howard Hughes Medical Institute, The Rockefeller University, NY, NY; the latter was supported by NSF and NIH. Both groups addressed critical issues in the avian literature. A persistent use of inappropriate terms for brain structures had occurred, based upon outdated assumptions of homology to mammalian structures, particularly in the forebrain that needed to be changed. I have kept in touch with Dr. Reiner and Dr. Jarvis over the years with issues that have surfaced regarding the use of appropriate terms and acronyms for avian neural structures.

In 1988 I published a book with Manju Masson titled ‘A Stereotaxic Atlas of the Brain of the Chick, *Gallus domesticus’* that showed the accurate location, name and its abbreviation (acronym) for all known neural structures throughout the brain of a chick. It was published by The Johns Hopkins University Press (Kuenzel and Mason, 1988). It was the first stereotaxic atlas of an avian brain displaying complete sets of images in three planes: coronal, sagittal and horizontal sections. A total of 500 copies of the book were produced by The Johns Hopkins University Press (TJHUP). Two or 3 years ago I received a letter from TJHUP that they released all rights to me and Manju for that book. I therefore copied the book and placed it on my website with the University of Arkansas and Scholarworks at the University of Arkansas. Individuals can download the book for no charge. To date, I was informed that 739 downloads have occurred. Currently I am working on a second edition of the stereotaxic atlas as the present publication is totally out-of-date. I am planning to complete the second edition with a colleague, Parker Straight, by the end of February 2026 with all known structures, their names, acronyms and specific locations.

The Third Academic process, listed as, (3) sabbatical years, in the previous, underlined sub-heading, significantly helped me during the years at UMD and UAF. Specifically, it was the granting of sabbatical departures for 1 year from each home university. I have taken four, 12-month sabbaticals and all were spent in a foreign country. The first occurred at the Roslin Institute, Edinburgh, Scotland. The funding of the sabbatical leave was supported by a Fulbright-Hayes Senior Research Fellowship. Five publications resulted: (1) Kuenzel (1982), Physiol. Behav. (2) Kuenzel and van Tienhoven (1982), J. Comp. Neurol. (3) Kuenzel (1983), Bird Behav. (4) Mass and Kuenzel (1983) Devel. Brain Res. (5) Kuenzel and Sharp (1985), British Poultry Sci.

The publication, (Kuenzel and van Tienhoven, 1982), stimulated me to start the 6-year process of developing our first stereotaxic atlas of the chick brain as it identified the accurate location of several hypothalamic nuclei and all circumventricular organs in the avian brain. A second publication revealed that parasagittal knife cuts that isolated the entire length of the hypothalamus from lateral neural connections resulted in a premature activation of the reproductive system. Data demonstrated that the surgical procedure produced increases in luteinizing hormone (LH) that significantly contributed to advancement of reproductive function, (Kuenzel and Sharp, 1985). Clearly the first sabbatical leave provided the stimulus for me to initiate developing a book, the stereotaxic atlas (Kuenzel and Mason, 1988).

The second sabbatical leave occurred at Justus Liebig University Giessen, Germany and was supported by a Fulbright-Hays Senior Research Fellowship. Professor Andreas Oksche was Chair of the Department of Anatomy and Director of a research center where I worked for a year that focused on research addressing extraretinal photoreceptors. Dr. Sabine Blӓhser was the professor whom I worked with at the center. Publications that occurred were: Kuenzel and Blӓhser (1991) and Kuenzel and Blӓhser (1994), both published in Cell and Tissue Research. The 1991 paper was the first to describe a complete distribution of gonadotropin-releasing hormone (GnRH) neurons and fibers throughout the avian brain, while the second described the distribution of vasoactive intestinal polypeptide (VIP) neurons, with an emphasis on the lateral septal organ. Dr. Oksche developed the concept of extraocular photoreception in birds that have photo-neuroendocrine cells. I named the lateral septal organ (LSO) in a previous publication (Kuenzel and van Tienhoven, 1982) and our lab showed that the LSO contains a number of cerebrospinal-fluid contacting VIP neurons as well as VIP receptors that appear to function as photoreceptors. The LSO has been proposed to be one of four locations within the avian brain that houses the appropriate neurons for the initial activation of reproductive function each year in avian species. To date, controversy continues regarding which of the four proposed structures is/are essential for this critical function or whether all work in some neural pathway to activate gonadal development seasonally.

The benefit of this sabbatical leave was documenting the complete distribution of gonadotropin-releasing hormone (GnRH) neurons within the brain of the chicken that initiate and maintain gonadal development throughout the lifetime of poultry. A second important neuron, the VIP neuron, occurs within the LSO. The VIP neurons are proposed to initiate seasonal reproductive function in developing chicks due to their response to increased photostimulation during the springtime and summer (Kuenzel and Blӓhser, 1994).

The third sabbatical was also located in Germany, in the Institute of Animal Welfare and Animal Husbandry, formerly named the Inst. of Animal Science and Behavior, Celle, Germany. The host was Dr. Roland Grossmann. Two papers were published in the J. Comp. Neurol. and both utilized the technique of *in situ* hybridization histochemistry (ISHH) gene expression (Kuenzel et al., 1997; Jurkevich et al., 1999. The firsr showed sites of gene expression of VIP throughout the brain of the chick. The second showed development of the sexually vasotocinergic cell type in the bed nucleus of the stria terminalis in chickens. ISHH was also utilized to document gene expression of GnRH-1 and VIP in neurons and compare the specific locations with previous data on the distribution of the peptides GnRH-1 and VIP using immunohistochemistry. The agreement was quite high with only a few additional sites of mRNA where we were unable to also see the peptide produced. The overall conclusion was to continue using results of immunohistochemistry to map specific locations of structures in the avian brain.

Our lab had shifted to neuroendocrine regulation of stress when I took a fourth sabbatical leave to the College of Veterinary Medicine, Nanjing Agricultural University, Nanjing, China. The host was Dr. RuQian (Lucy) Zhou. An experiment in our lab displayed a significant sex difference in the stress response between roosters and hens. Specifically, a significantly greater amount of stress hormone, corticosterone, was released following administration of equivalent doses of either the major neural hormone corticotropin releasing hormone (CRH) and/or arginine vasotocin (AVT) in undisturbed birds (Madison et al., 2008). We discovered for the first time an additional neural structure that plays an important role in the stress response of birds, the nucleus of the hippocampal commissure (NHpC). Of interest, the NHpC is located in the septum, a region dorsal to the hypothalamus (Nagarajan et al., 2014; Nagarajan et al., 2017a; Nagarajan et al., 2017b; Nagarajan et al., 2017b made the cover of the Journal of Neuroendocrinology (J. Neuroendo.) and a podcast was produced on its website following an interview of the Editor of J. Neuroendo. with the senior author, Gurueswar Nagarajan. Dr. Nagarajan had previously received a Ph.D. in my lab and at the time of the interview was a visiting NIH Fellow. Currently he is a scientist at the Henry M. Jackson Foundation for the advancement of Military Medicine in Bethesda, MD. A substantial population of CRH neurons were found in the NHpC and were significantly larger than the classical CRH hypothalamic neurons, specifically in the paraventricular (PVN) nucleus, the major site for the stress response in mammals.

Subsequent experiments examining two very different stressors: (1) food deprivation, a stressor that gradually increases in strength with time due to the absence of essential nutrients when food is withdrawn and (2) immobilization, a stressor that is immediately stressful as each bird was placed in a harness that reduced its movement of wings and legs. Regardless of the stressor, the same sequence of gene expression occurred. In summary, using immobilization stress, the sequence of gene expression was (1) a peak of CRH gene expression within the NHpC, located in the septum, (2) a peak of CRH gene expression within the paraventricular (PVN) hypothalamic nucleus, (3) a peak of the peptide hormone, arginine vasotocin (AVT) in the PVN that sustained the stress response at the level of the brain (Kadhim et al., 2021) and also included receptors of CRH and AVT in the two brain structures as well as measured gene expression in the anterior pituitary.

### University service

For the past 28 years, I have served as the University of Arkansas Representative and previously the University of Maryland Representative for the USDA Regional Project NC-1170 Advanced Technologies for the Genetic Improvement of Poultry. In 2018, I organized a mini-symposium with Dr. Hans Cheng entitled ‘Current Interest in Gene Editing of Avian Species’ during an afternoon of the Poultry Workshop supported in part by USDA Regional Project, NC-1170. Eight prominent speakers were invited who were recognized for their knowledge of gene editing. The workshop was well attended at the Plant and Animal Genome (PAG) meeting in San Diego, CA. All seats were taken throughout the entire symposium with standing room only for all speakers.

Served for 10 years (2013–2023) on the Joint Patent and Copyright Committee and was Chairman for 5 years (January 2018 to December 2023.

To date, I have had the pleasure of serving as major professor of 27 graduate students who successfully pursued a Master of Science, MS degree and 13 doctoral students who completed their PhD program. Funding of the students occurred using several programs. Primary ones included the University of Maryland and University of Arkansas at the Dept., College and Univ. levels, including the Agricultural Experiment Station, National Science Foundation (NSF), USDA Competitive Grants Program, National Institutes of Health (NIH), Arkansas Biosciences Institute (ABI) Grants, Chancellor’s Innovation and Collaborative Fund and company grants programs.

Publications include: 126 Journal Papers, 1 Book, 11 Chapters in Books, 206 Abstracts from Scientific Society Meetings, 51 Papers at Conferences, Short courses or Workshops, 3 articles in Trade or Popular Press sources, and 2 Patents in which one was licensed by a company. Currently I am working on a book, the Second Edition of ‘A Stereotaxic Atlas of the Brain of the Chick, (*Gallus gallus domesticus*)*.* The authors are Parker Straight and Wayne Kuenzel and is planned for completion in 2026.

## Awards

1. Sept. 1971-September 1973 National Institutes of Health (NIH) Postdoctoral Fellow, Cornell University, Ithaca, N.Y.
2. Jan. 1981 - Jan. 1982 Fulbright-Hays Senior Research Fellowship to Great Britain, Roslin Institute, Edinburgh, Scotland (Sabbatical Year #1). Hosts: Dr. Ian Duncan and Dr. Peter Sharp.
3. 1983 Maryland Alumni Assn (MDAA) Award, College of Agriculture for Excellence in Research.
4. 1988 Excellence in Teaching Award, Poultry Science Assn, Embrex (Purina Award), presented to an individual each year.
5. Aug. 1988 - Aug. 1989 Fulbright-Hays Senior Research, Fellowship to West Germany, Justus Liebig University Giessen (Sabbatical Year #2). Host: Dr. Sabine Blahser.
6. Aug. 1996 – Aug. 1997 Institute of Animal Science and Behavior, Celle, Germany (Sabbatical Year #3). Host: Dr. Roland Grossmann; Residence for family provided.
7. 1998 Merck and Co. Award for Achievement in Poultry Science, Poultry Science Association.
8. 2000 25 Years of Service Award Univ. of Maryland, Poultry Science Dept.
9. 2000 Sigma Xi Research Award-Contribution to Science Award, Univ. of Mayland Chapter.
10. 2010 Embrex Fundamental Science Award: Outstanding achievement in basic disciplines, Poultry Science Association.
11. 2012 Elected Fellow, Poultry Science Association.
12. September 2014-June 2015 Key Lab ANSC/BioChem, College VetMed, Nanjing Agricultural University, Nanjing, China (Sabbatical Year #4). Host: Dr. RuQian (Lucy) Zhou.
13. 2023/24 Elected Fellow, American Association for the Advancement of Science (AAAS).
14. 2025 25 Years of Service Award Univ. of Arkansas, Poultry Science Dept.
